# Guideline Implementation for Improved Asthma Management and Treatment Adherence in Children in Jordan

**DOI:** 10.3390/healthcare11121693

**Published:** 2023-06-09

**Authors:** Roqia Maabreh, Mahmoud H. Alrabab’a, Madiha Amin Morsy, Hekmat Yousef Al-Akash, Ahmad Rajeh Saifan, Nabeel Al-Yateem

**Affiliations:** 1 Faculty of Nursing, Al-Balqa Applied University, Alsalt 19117, Jordan; 2 Pediatric Nursing Department, Faculty of Nursing, Ain Shams University, Cairo 11517, Egypt; 3 Faculty of Nursing, Applied Science Private University, Amman 11937, Jordan; 4 College of Health Sciences, University of Sharjah, Sharjah 27272, United Arab Emirates

**Keywords:** asthma, management, adherence, experimental study, Jordan

## Abstract

Asthma imposes a significant social burden on children, their families, and society at large. As a chronic health condition, effective management could greatly benefit from consistent adherence to guidelines. Despite this, minimal effort has been exerted to examine the impact of asthma management guidelines and adherence to treatment on children with asthma and the mothers caring for them. This study was designed to evaluate the influence of asthma management guidelines on the knowledge and treatment adherence among children with asthma and their mothers. A quasi-experimental design was utilized, and the study was conducted at two large hospitals in Jordan: Princess Rahma Hospital and King Hussein Medical Center. A purposive sample of children aged 6–12 years (*n* = 100) who were accompanied by their mothers (*n* = 100) were recruited for this study. Data were collected using a structured questionnaire and an observation checklist before and after the implementation of guidelines. Statistical analyses were performed using SPSS. The results revealed a statistically significant improvement in knowledge related to asthma among children and their mothers (*p* < 0.001). Additionally, a statistically significant difference was observed in the children’s adherence to their treatment regimen before and after the implementation of asthma management guidelines (*p* < 0.001). Moreover, the improvements in knowledge and practice related to asthma were sustained in the follow-up assessments. In conclusion, the guidelines had a beneficial effect on the children’s adherence to their treatment regimen both before and after their implementation. Thus, asthma patients should adhere to conventional guidelines at various health services to manage their condition effectively.

## 1. Introduction

Asthma is a chronic inflammatory disorder of the airways, causing recurrent episodes of wheezing, dyspnea, chest tightness, and cough. It is usually associated with variable airflow obstruction, often reversible either spontaneously or with treatment [1,2]. The disease is mediated through many cells and cellular elements, particularly mast cells, eosinophils, T lymphocytes, macrophages, neutrophils, and epithelial cells, along with an associated increase in tracheal and bronchial hyper-responsiveness (BHR) to a variety of stimuli [3,4,5,6].

Two categories of predisposing factors have been documented: genetic and environmental factors [7,8,9,10]. Environmental factors include exposure to air pollution and allergens, smoking during pregnancy, and the use of some medications such as aspirin and beta blockers [2]. Genetically, many of the asthma-related genes relate to the immune system or participate in inflammation. Even among this list of genes supported by highly replicated studies, results have not been consistent across all populations [11]. Some studies have also reported that emotional and behavioral disturbances are more closely related to poor control of asthma than to the severity of the attack itself [12].

Many other health conditions also occur alongside asthma, including gastro-esophageal reflux disease (GERD), rhino-sinusitis, and obstructive sleep apnea [13]. Other researchers have also reported cases of psychological disorders, such as anxiety and mood disturbances [13,14,15].

Asthma is common among children, representing a significant burden on them, their families, and the community. It is estimated that asthma affects between 1 and 18% of children in developing countries, and between 11 and 20% in developed countries [2]. Overall, asthma presents a serious public health problem globally. The economic burden is tied to the high cost of hospitalization due to the recurrent nature of the disease [16]. Another significant burden is children’s absence from school [17]. Studies show that asthma accounts for more school absences and hospitalizations than any other chronic condition [18]. In Egypt, up to one in four children with asthma is unable to attend school regularly because of poor asthma control, and increasing cases of morbidity and mortality have also been reported [19].

The prevalence of asthma varies with time. As of 2011, 235–330 million people worldwide were affected by asthma [2,20,21], and approximately 250,000–345,000 children die per year from the disease [2,20]. More cases have been reported among children than adults [22]. According to Centers for Disease Control and Prevention (CDC) data, asthma affects approximately 8.5% of the pediatric population in the United States of America (USA), or more than 7 million children. The prevalence is 8–10 times higher in developed countries, such as the United States, Great Britain, Australia, and New Zealand, than in developing countries, such as in Southeast Asia. In Jordan, there has been a twofold increase in the prevalence of asthma in the last 10 years [23]. A study reported that the prevalence of asthma among adolescents aged 13 to 14 years in Irbid City, Jordan was 12.3% [24].

Currently, the diagnosis of asthma relies on many findings, with clinical exams primarily depending on the pattern of symptoms and response to therapy over time [25]. However, the diagnosis of asthma should be suspected if there is a history of recurrent wheezing, coughing, or difficulty breathing, and if these symptoms occur or worsen due to exercise, viral infections, allergens, or air pollution. Spirometry is then used to confirm the diagnosis [26]. The American Thoracic Society has provided strategies in its information series for patient education about how children can successfully perform pulmonary function tests [26]. However, the European Respiratory Society (ERS) recommends spirometry as a first-line diagnosis in children aged 5–16 years with suspected asthma and bronchodilator reversibility testing as a first-line diagnosis in all children with forced expiratory volume 1(FEV) < 80% predicted [27]

For asthma management, the GINA latest guidelines of the year 2022 recommends the use of low-dose inhaled corticosteroids (ICSs) with short-acting beta agonists (SABAs) or very low doses of ICS-formoterol maintenance and reliever (MART) to control symptoms and prevent exacerbations [28]. Moreover, avoidance of triggers is a key component of improving control and preventing attacks. Conventional guidelines exist to assist in managing symptoms. The National Asthma Education and Prevention Program (NAEPP) and National Heart, Lung, and Blood Institute (NHLBI) have provided guidelines for improving the quality of clinical healthcare [29]. Further evidence has shown that guideline reminders for healthcare providers (HCPs) are an effective intervention to increase patient adherence [30]. Healthcare providers often rely on providing health education and delivering information about management guidelines and adherence to the recommended guidelines for mothers when their children are underage. Despite evidence that mothers’ interventions may assist in improving children’s adherence to recommended guidelines, little has been conducted to examine the impact of implementing improved asthma management and treatment adherence guidelines among children in Jordan. Thus, this study was conducted to fill this knowledge gap.

## 2. Methods

### 2.1. Study Design

A quasi-experimental design was utilized in this study to examine the impact of asthma management guidelines on the practices of mothers and children in managing asthma and monitoring disease progress.

### 2.2. Study Setting

The study was conducted at two large hospitals in the two largest cities in Jordan, Amman (the capital of Jordan) and Irbid (in the northern region of Jordan). These hospitals were randomly selected after clustering hospitals based on the two geographical regions of Jordan.

### 2.3. Ethical Approval

Before the study commenced, ethical approval was sought and obtained from the scientific research ethical committee in the faculty of nursing at Applied Sciences Private University, Amman, Jordan (Ref # Faculty-2019-2020-1-2). Written informed consent were given by all participants (mothers on behalf of themselves and their children). The confidentiality and privacy of the participants and data were maintained. Participants’ personal information (i.e., names) were coded and saved in a coding sheet that is accessible only to the research team and no identifiers were used.

### 2.4. Sample and Sampling

A purposive sample of children aged 6–12 years (*n* = 100) who were accompanied by their mothers (*n* = 100) were recruited for this study. The recruited children were previously diagnosed with asthma by their physicians, as documented in their medical records, and were among the outpatient attendees at the healthcare center where they were accompanied by their mothers. At this age, children’s cognitive abilities might be insufficient to help them understand the intricacies of self-care or adhere to a proper medication regimen. Mothers often take care of their children, and thus they were also included in the study. Moreover, due to cultural considerations, mothers (not fathers) often accompany their children, especially if they are admitted to the pediatric wards. Male companions are usually not allowed in order to maintain mothers’ privacy, especially at night, and to avoid social conflicts.

Asthma diagnoses were based on the 2019 GINA guidelines [31]. The severity of asthma was assessed using the same 2019 GINA guidelines, as the level of treatment required the control of symptoms and exacerbations. Mild asthma is controlled with low doses of inhaled corticosteroids (ICSs), while severe asthma requires high doses of ICSs [31].

### 2.5. Data Collection

The study began in July 2019 and proceeded until July 2020. Data were collected from selected participants in different phases by researchers who received comprehensive training from the principal investigator on how to use the instruments for data collection and how to conduct the intervention sessions. Data were gathered before and after the implementation of the developed guidelines.

Two different tools were used to collect the data. First, a 3-part questionnaire, which was developed by the researchers after an extensive review of the literature, was used to assess the children’s and mothers’ knowledge about asthma. The first part included the children’s and mothers’ sociodemographic data and other information about them, such as the history of the disease and the number of attacks. The second part assessed the children’s and mothers’ knowledge about asthma, involving 15 multiple-choice questions on topics such as the definition, types, predisposing factors, etiology, and steps for asthma treatment regimens. A score of “2” was assigned for a “correct” answer, “1” for an incomplete answer, and “0” for an incorrect answer. A cut-off point of 60% was set, with a knowledge percentage of 60% or above considered as “satisfactory” and less than 60% as “unsatisfactory”. The developed tool was revised and evaluated by three experts in the pediatric nursing field for its applicability, feasibility, and clarity.

Second, an observation checklist with 12 items was used to assess the actual management of children’s diseases through the use of metered dose inhalers (MDIs). This checklist, adopted from the literature [32], was revised and evaluated by three experts in the pediatric nursing field for its applicability, feasibility, and clarity. Both tools were then piloted to test their reliability and validity and were found to be valid and reliable. Responses to the items in the checklist were categorized as done or not done.

### 2.6. Study Intervention

The asthma management guidelines were designed by the researchers in accordance with the 2019 GINA guidelines [31]. These were structured to be clear and comprehensible to the layperson. The guidelines covered the following areas: an overview of asthma as a disease; its definition, levels, and severity; triggering factors for attacks; signs and symptoms of asthma; complications; diagnostic measures; preventive measures to limit disease exacerbations; therapeutic modalities (pharmacologic and non-pharmacologic); and the proper use of drugs to control asthma. The asthma management intervention program was then revised and approved by experts in asthma management. The program was implemented over five sessions, each an hour long, in the healthcare center’s seminar room. The administration of the program included the provision of relevant information through PowerPoint presentations, in addition to demonstrations and videos about how to use asthma medications to make the ideas clearer for the children and their mothers. Finally, participants were encouraged to take the opportunity to ask questions.

### 2.7. Data Analysis

After the intervention, the researcher recollected data in the evaluation phase using the same pre-test format to assess the impact of the asthma management guidelines on the knowledge of children with asthma and their mothers. The data were then analyzed using SPSS tools, Chi-squared tests, and descriptive statistical tools. Statistical significance was considered at *p* < 0.05 and high significance at *p* < 0.001.

## 3. Results

Regarding the demographic characteristics of the children, there were more boys (58%) than girls (42%) in the study. It was also observed that 42% of the studied children were in the age group of 8 to 10 years, with a mean age of 8.52 (SD = 1.45). The majority of the sample was in the first and third grades, each making up 33% of the total. Table 1 presents the characteristics of the studied children.

Regarding the children’s mothers, most of them were between the age of 30 and 35 (40%). It was also recorded that 60% of the mothers had secondary school level education. Table 2 present the characteristics of the studied children’s mothers.

This study also gathered data about the children’s history of illness. Accordingly, it was noted that 58% of the studied children developed asthma between one and three years before the time of the study, while 42% developed the condition before one year. Moreover, it is noted from the table below that 58% of the children were discovered to have asthma through their regular checkup where they intentionally came to seek medical care for respiratory symptoms, whereas 42% were accidentally discovered to have asthma. Table 3 represents the history of the Asthma disease among participants.

**Figure 1:**
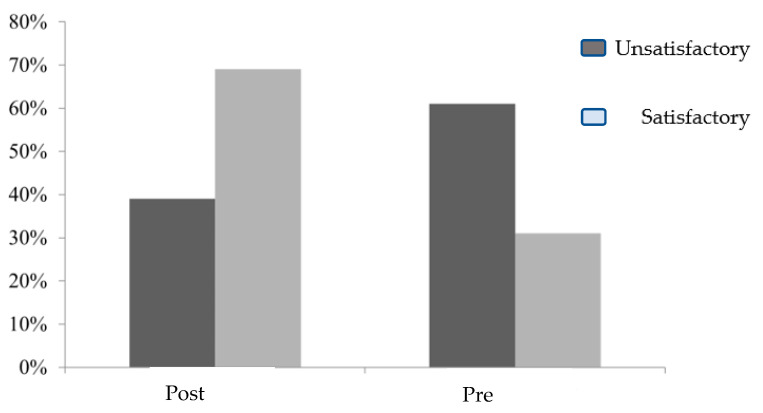
Percentage distribution of the studied children according to total knowledge score pre-test and post-test regarding knowledge about asthma.

The children’s knowledge about asthma was assessed before and after the implementation of the guidelines. The bar graph below shows an improvement in children’s knowledge from pre-test to the post-test following the implementation of the guidelines (Figure 1). From the figure below, it is noticeable that there was an improvement in children’s knowledge from pre-test to post-test following the implementation of the guidelines.

The children’s knowledge score levels in the pre- and post-tests regarding triggering factors for asthma were also analyzed. From this analysis, it was observed that there was a statistically significant difference between the children’s knowledge scores in the pre- and post-tests concerning triggering factors for asthma (*p* < 0.05) (Table 4). The differences were apparent in various concepts, including dust, pesticides, flowers, and plants.

The children’s knowledge levels regarding signs of asthma were also assessed in the pre- and post-tests using a questionnaire. It was noted that there was a statistically significant improvement in knowledge scores before and after the implementation of the management guidelines (*p* < 0.05). The difference was observed in many signs of asthma, including tachycardia, dyspnea, and the recognition of common cold symptoms (Table 5).

The children’s knowledge score levels in the pre- and post-tests regarding preventive measures to avoid asthma were also assessed and reported (Table 6). It was noted that there was a statistically significant difference between the knowledge score levels of the children studied in the pre- and post-tests concerning measures to prevent asthma (*p* < 0.05). Some of the key preventive measures included avoiding strong-smelling substances, such as perfume and cleaning materials.

The children’s knowledge score levels in the pre- and post-tests regarding reported practice treatment of asthma were also assessed, and a statistically significant difference was found between the knowledge score levels of the children studied in the pre- and post-tests regarding reported practice treatment of asthma (*p* < 0.05) (Table 7).

**Figure 2:**
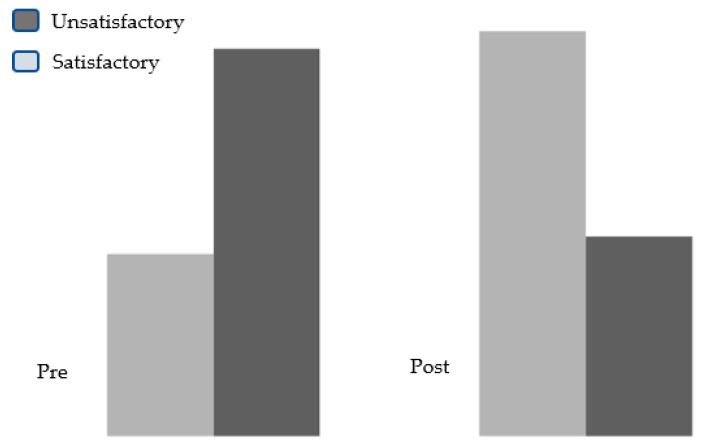
Percentage distribution of the studied children according to knowledge pre- and post-test regarding knowledge about medication of asthma.

The children’s knowledge in the pre- and post-tests regarding asthma medication was also analyzed, and an observable improvement was noted in the post-test concerning asthma medications (Figure 2).

**Figure 3:**
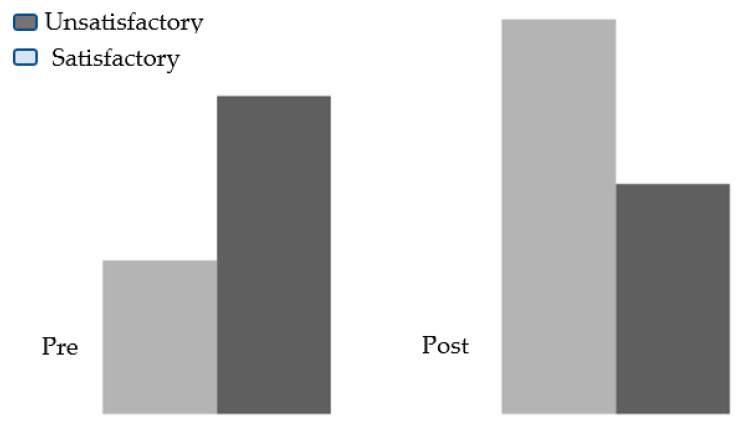
Percentage Distribution of the Studied Children According to Knowledge Pre- and Post-test Regarding Use Bronchodilators Asthma.

There was also a significant difference between the children’s knowledge practice scores in the pre- and post-tests regarding the use of bronchodilators for asthma (Figure 3). The difference indicates a positive impact from the implementation of asthma management guidelines.

The actual knowledge and practices of children and their mothers regarding asthma drugs pre- and post-instructional guidelines were assessed. The outcomes showed that there was a statistically significant difference in knowledge and practices related to using asthma drugs (*p* < 0.05). Table 8 presents the details of this distribution.

This study further assessed children’s progress in follow-up studies through five repeated measures regarding their frequency of asthma attacks and the severity of asthma symptoms. Accordingly, there was a significant improvement, consistent across the five measurements (Table 9).

The assessment of children according to adherence to inhaler use showed an overall statistically significant improvement in the level of adherence to the inhalers (*p* < 0.001) (Table 10).

## 4. Discussion

Asthma is a chronic condition that often presents debilitating symptoms among children, thus necessitating consistent care and management. This study examined children’s knowledge and some practices regarding asthma. The preliminary observations indicated that the onset of asthma typically occurs within the first five years of life. This finding concurs with Dowell [33], who noted the same result among their study participants. This outcome can be attributed to a combination of environmental and hereditary factors.

Concerning the overall knowledge of the study sample, this study observed a significant improvement in total knowledge about asthma after the intervention. This finding aligns with the conclusion of Szefler et al., who reported that asthma education guidelines applied to children and their mothers could succeed in improving concepts and knowledge, as well as fostering significant self-management behaviors [17]. Moreover, the majority of asthmatic children and their mothers had unsatisfactory knowledge about asthma prior to the intervention. However, there was a satisfactory level of knowledge after the intervention. These results reflect the significant impact that the management guidelines had among the participants.

Regarding the signs and symptoms of asthma, the present study revealed a significant improvement in children’s knowledge in the post-test scores. These results indicate that the guidelines had a positive effect on the knowledge of children and their mothers. These findings align with Zhao et al., who discovered that parents lacked awareness of the clinical manifestations of asthma and the indicators of acute attacks [34]. Thus, mothers often have an understanding of the common symptoms of asthma among their children, but not of all the signs and symptoms.

With respect to the knowledge of children and their mothers about triggering factors of asthma, this study showed that children’s knowledge regarding triggering factors for asthma, such as dust and smoke, flowers, pesticides, birds, and animals, was significantly higher in the post-test than in the pre-test. This outcome is underscored by Biksey et al. [35], who conducted a pilot study of asthmatic children, their parents, and home environments and reported that house dust, mites, molds, and smoke are significant triggers of asthma. However, most mothers did not have comprehensive knowledge about the triggers of asthma. Some studies have also reported that most mothers have adequate knowledge about the triggers of asthma [36].

Concerning the knowledge scores of the study sample about the complications of asthma, it was observed that there was a significant improvement in children’s and their mothers’ knowledge in the post-test compared to the pre-test regarding the complications of asthma. This finding was consistent with Morton et al., who reported significant improvements in participants’ knowledge upon the introduction of an intervention program [37]. The same improvement was noted among children and mothers’ knowledge about factors to avoid. These improvements can be attributed to the intervention program. Nevertheless, previous studies have also reported a significant improvement in knowledge regarding preventive measures to avoid. Evidence indicates that more than half of the mothers mentioned incomplete information about allergens and irritants to prevent an asthma attack. However, children and mothers generally knew that by avoiding asthma triggers and irritants, they could reduce the risk of an asthma attack. Concurrently, Al-Binali et al. [36] reported that all mothers had the idea that regular administration of necessary medication would prevent asthma attacks.

Better knowledge about the causes of an asthma attack was disproportionate to two main triggers, including allergens and irritants. This could be due to the past history of encountering these two triggers only. Overall, the total mean scores of children’s and their mothers’ knowledge about definition, symptoms, prevention of triggers, and management of asthma significantly improved in the post-test compared to the pre-test, which can be attributed to the implemented guideline.

The impact of guidelines on children’s and their mothers’ practices regarding asthma treatment was also assessed. This study found that there was a significant improvement in practice after implementing the guideline. Consequently, more mothers reported giving prescribed medication to their children in the post-test compared to the pre-test. The improvement in practice is consistent with the reports established by Brown et al. (2010), who noted improvements in medication administration and clinic attendance in only half of the participants [38]. According to Al-Binali et al. [36], the majority of mothers responded by giving medications and seeking medical attention during asthma attacks.

Regarding the medication dose in the nebulizer session, the majority of mothers followed the doctor’s prescription. This adherence to dosage might be related to mothers’ efforts to avoid complications. Additionally, most mothers consider giving an accurate dose of medication in the nebulizer as the most important step in controlling and managing an attack.

Moreover, this study observed that the majority of mothers check the inhaler for the presence of dust, shake the inhaler well, ask the child to breathe out, ask the child to take 6–7 breaths after raising the puff, and clean the spacer or cover the inhaler after removing it from the child’s mouth. However, this observation contradicts the findings of Miller et al. [7], who discovered that more than two-thirds of mothers had poor practice regarding the use of the inhaler. It is common for parents of children with persistent asthma to have inappropriate use of inhaled corticosteroid therapy. Poor practices regarding inhaler use could arise from the complicated nature of the device, which requires detailed instructions for correct usage.

This study also noted a significant association between mothers’ level of education and their practices regarding asthma management. The literature indicates that there is a statistically significant relationship between care management score and mothers’ level of education [38]. However, Tantawi et al. [39] reported that there was no significant difference between mothers’ practices and their socio-demographic characteristics, such as age and education. Nevertheless, educated mothers have a better chance of gaining knowledge from different sources and applying it in asthma management compared to non-educated mothers. Some studies have also noted a significant impact of mothers’ moods, knowledge, and their practices of asthma management.

However, the relationship between knowledge and reported behavior in childhood asthma management is nonlinear. These results suggest that knowledge about asthma can influence behavior, but only under certain conditions. It can be interpreted that mothers with knowledge about asthma voluntarily decide when to practice it and when to disregard it.

## 5. Limitations

While some of the observations made in this study concur with many previous research observations, readers need to consider that the study was conducted among a smaller sample size of 100 children and 100 mothers. This was based on their availability and consent. Therefore, the outcomes are limited to a smaller sample. Moreover, this study did not have a parallel control group to compare the effects of treatments.

## 6. Conclusions

This study focused on assessing the impact of asthma management guidelines on children and mothers’ knowledge and adherence to asthma management. Through the quasi-experimental approach, the study showed a significant improvement in knowledge and practices of asthma management after implementing the management guidelines. Therefore, this study recommends the utilization of asthma management guidelines at various health services to overcome the challenges of asthma. Nevertheless, further research is needed among a relatively larger sample size and in different settings for comparative observations. Moreover, qualitative inquiry is highly recommended to better understand mothers’ experiences of parenting asthmatic children and to understand children’s experiences and perspectives concerning their asthma diagnosis.

## Figures and Tables

**Table 1:** Characteristics of the Studied Children (N = 100).

Variable	Frequency	Percentage
Age		
6–<8	41	41.0
8 < 10	42	42.0
10 ≤ 12	17	17.0
Mean (SD)	8.52 (1.45)	
Gender		
Boys	58	58.0
Girls	42	42.0
Grade	No	%
First grade	33	33.0
Second grade	8	8.0
Third grade	33	33.0
Fourth grade and above	26	26.0

**Table 2:** Characteristics of the Studied Children’s Mothers (N = 100).

Age	Frequency	Percentage
Age		
25 < 30	34	34.0
30 < 35	40	40.0
35 < 40	17	17.0
40+	9	9.0
Mean (SD)	32.5 (4.69)	
Educational level		
Secondary	60	60.0
University	40	40.0

**Table 3:** History of the disease of asthma.

History of Disease	Frequency	Percentage
Duration of illness (year)		
Less than 1 year	42	42.0
Between 1 and 3 years	58	58.0
Mean (SD)	1.08 (0.5)	
Appearance of the disease		
Regular checkups	58	58.0
Accidentally	42	42.0

**Table 4:** Distribution of the studied children according to their knowledge score level pre-test and post-test about triggering factors for asthma (N = 100).

Factors	Pre-test	Post-test	χ²	*p*
Throat inflammation and tonsils	50	67	5.95	0.01
Dust	85	94	4.31	0.05
Vapors and smoke	89	96	3.53	0.12
Flowers and plants	81	92	5.18	0.05
Pesticides	81	94	7.82	0.01
Feathering birds and animals	33	89	65.91	0.01
Sniff aromatherapy and perfumes	79	89	3.72	0.17
Material petroleum and gases	80	91	4.88	0.05
High-percentage humidity	19	84	84.58	0.01
Exposure to air currents	39	79	33.07	0.01
Take some medication	16	73	65.78	0.01
Psychological situation	41	81	33.63	0.01
Extreme effort	82	91	4.89	0.01
Take some foods	41	81	33.63	0.01

**Table 5:** Distribution of the studied children’s knowledge score level pre-test and post-test regarding signs of asthma (N = 100).

Sign	Pre-Test	Post-Test	χ^2^	*p*
Bluish color of the nails and lips	31	74	35.41	0.01
Tachycardia	52	83	20.68	0.01
Dyspnea	80	96	16.61	0.01
A sense of common cold symptoms	32	79	42.94	0.01
Feeling tired	84	91	9.07	0.01
Sleep disorder	57	77	8.23	0.01
All of the above	26	72	40.40	0.01

**Table 6:** Distribution of the studied children by their knowledge score level pre-test and post-test regarding preventive measures to avoid asthma (N = 100).

Action	Pre-Test	Post-Test	χ²	*p*
Avoid sudden exposure to different temperatures	57	83	16.09	0.01
Stay away from people infected with influenza	57	86	20.64	0.01
Avoid vigorous exercise	91	94	0.00	
Avoid using strong-smelling substances such as perfume and cleaning materials	69	91	15.14	0.01
Avoid the child’s exposure to the fumes and dust	79	94	22.14	0.01
Wear warm clothing to protect against the cold.	41	76	25.23	0.01
Avoid exposure to cold air drafts after being in a warm area	36	74	29.17	0.01
Avoid foods and drinks that cause asthma	26	86	73.05	0.01
Use of various breathing exercises	31	66	24.52	0.01
Taking the medication prescribed by the child’s doctor’s instructions	100	100		

**Table 7:** Distribution of the studied children by their knowledge score level pre- and post-test regarding reported practice treatment of asthma (N = 100).

Remedial Action	Pre-Test	Post-Test	χ²	*p*
The practice of various breathing exercises	29	66	27.45	0.01
Supplying oxygen by doctor’s orders	49	89	37.40	0.01
Vapor inhalation according to doctor’s orders	52	87	28.89	0.01
Taking the medication prescribed by the doctor	89	98	11.41	0.01
Use asthma medications as per doctor’s orders	85	98	10.86	0.01
Using nebulizer as per doctor’s orders	56	82	15.80	0.01
Calming and reassuring the child and reduce fear	50	82	22.82	0.01
Moving the child to a well-ventilated place	71	86	6.67	0.01
Child sits while the head is lifted up	34	74	32.21	0.01
Give the child any kind of herbs or popular descriptions (natural remedies)	50	30	8.33	0.01
Going immediately to the specialized doctor or hospital	54	91	34.33	0.01

**Table 8:** Distribution of children and their mothers’ actual knowledge and practice regarding asthma drugs pre- and post-instructional guideline.

	Pre		Post		χ²	*p*
	Satisfactory	Unsatisfactory	Satisfactory	Unsatisfactory		
	%	%	%	%		
Knowledge	46	54	82	18	28.2	0.01
Practice	56	44	80	20	13.2	0.05

**Table 9:** Frequency of asthmatic attacks and severity of asthma symptoms.

Item	Baseline	After 1 Month	After 3 Months	After 6 Months	After 12 Months
	%	%	%	%	%
Frequency of asthmatic attacks/month					
Once	24	24	27	31	61
Twice	42	42	44	53	36
Three times	26	26	23	12	3
≥Four times	8	8	6	4	0
Severity of asthma symptoms					
Mild	36	36	41	50	63
Moderate	38	38	35	37	37
Sever	26	26	24	13	0

**Table 10:** Distribution of children according to adherence to inhalers’ utilization pre- and post-test.

Item	Pre		Post		χ²	*p*
	No	%	No	%		
Utilization of Inhaler						
Satisfied	52	52	86	86	27.1	<0.001
Un Satisfied	48	48	14	14		

## Data Availability

Data can be provided upon reasonable request made to the PI.
